# Ethical Considerations in Ever-Expanding Utilization of ECLS: A Research Agenda

**DOI:** 10.3389/fped.2022.896232

**Published:** 2022-05-19

**Authors:** Jonna D. Clark, Harris P. Baden, Emily R. Berkman, Erica Bourget, Thomas V. Brogan, Jane L. Di Gennaro, Ardith Z. Doorenbos, D. Michael McMullan, Joan S. Roberts, Jessica M. Turnbull, Benjamin S. Wilfond, Mithya Lewis-Newby, Seattle Ethics in ECLS (SEE) Consortium

**Affiliations:** ^1^Division of Pediatric Critical Care Medicine, Department of Pediatrics, University of Washington School of Medicine, Seattle, WA, United States; ^2^Division of Pediatric Bioethics and Palliative Care, Department of Pediatrics, University of Washington School of Medicine, Seattle, WA, United States; ^3^Treuman Katz Center for Pediatric Bioethics, Seattle Children's Research Institute, Seattle, WA, United States; ^4^Fred Hutchinson Cancer Research Center, University of Washington School of Medicine, Seattle, WA, United States; ^5^Department of Anesthesiology and Pain Medicine, University of Washington School of Medicine, Seattle, WA, United States; ^6^Department of Biobehavioral Nursing Science, College of Nursing, University of Illinois, Chicago, IL, United States; ^7^Division of Pediatric Cardiothoracic Surgery, University of Washington School of Medicine, Seattle, WA, United States; ^8^Division of Pediatric Critical Care Medicine, Department of Pediatrics, Vanderbilt University Medical Center, Nashville, TN, United States; ^9^Center for Biomedical Ethics and Society, Vanderbilt University Medical Center, Nashville, TN, United States

**Keywords:** critical care, ethics, extracorporeal life support (ECLS), extracorporeal membrane oxygenation (ECMO), research, communication

## Abstract

Technological advancements and rapid expansion in the clinical use of extracorporeal life support (ECLS) across all age ranges in the last decade, including during the COVID-19 pandemic, has led to important ethical considerations. As a costly and resource intensive therapy, ECLS is used emergently under high stakes circumstances where there is often prognostic uncertainty and risk for serious complications. To develop a research agenda to further characterize and address these ethical dilemmas, a working group of specialists in ECLS, critical care, cardiothoracic surgery, palliative care, and bioethics convened at a single pediatric academic institution over the course of 18 months. Using an iterative consensus process, research questions were selected based on: (1) frequency, (2) uniqueness to ECLS, (3) urgency, (4) feasibility to study, and (5) potential to improve patient care. Questions were categorized into broad domains of societal decision-making, bedside decision-making, patient and family communication, medical team dynamics, and research design and implementation. A deeper exploration of these ethical dilemmas through formalized research and deliberation may improve equitable access and quality of ECLS-related medical care.

## Introduction

“*Almost every action within the medical setting either explicitly or implicitly contains two judgements, one ethical and one scientific, and there is constant interplay between what is technically possible and what is morally desirable”* (1).

Although traditionally used in neonatology and pediatric medicine for acute respiratory failure, extracorporeal life support (ECLS) is now the standard of care for an expanding number of pediatric and adult medical conditions. ECLS is commonly deployed to allow recovery from potentially reversible conditions, such as acute respiratory distress syndrome (ARDS) and shock; as a bridge to decision, when prognosis is uncertain; as a rescue therapy during cardiopulmonary arrest that is unresponsive to initial resuscitation measures; and as a bridge to transplant or destination therapy with mechanical circulatory support (2–5). Over the last decade, exponential growth of ECLS utilization in adults (6, 7) has surpassed its utilization in children. As of March 2022, 92,389 adults and 79,604 children (age <18 years of age) were supported by ECLS with survival rates ranging from 49% (adults) to 65% (neonates) (8). The COVID-19 pandemic likely contributed to this expansion in the adult population, as 13,341 patients with ARDS secondary to SARS-CoV-2 infection have been managed with ECLS (8–10).

Rapid technological advancements and the exponential expansion of ECLS utilization, as exemplified by the SARS-CoV-2 pandemic, are generating an increasing number of ethical and societal considerations (11–16). Clinical and ethical guidance for the utilization of ECLS are often based on expert consensus, case series, and retrospective cohort analyses due to inherent challenges in prospective outcomes studies and randomized control trials in these populations (17, 18). Over the last decade, clinical guidelines (9, 19–21), position and opinion papers (2, 22–26), surveys of clinician perceptions (27–31), and discourse surrounding ethical considerations (32–36) have proliferated. Notably, explicit discussions regarding ethical considerations are often implied or excluded from these clinical guidelines, position statements, and surveys of clinician perceptions. Furthermore, thoughtful and detailed manuscripts that explicitly highlight general ethical principles often do not provide practical guidance for clinicians. Hence, these limitations in the current medical literature compel further exploration of complex ethical questions through additional empirical and normative research (2). Failure to address ethical implications associated with complex decision making in ECLS increases the risk of unnecessary and undesired burdens for patients, families, staff, and health care systems (37). In addition, institutional, regional and global variation in ECLS application may exacerbate already existing health care disparities (27, 38, 39).

## Brief History and Description of ECLS

Since its introduction nearly fifty years ago, ECLS has provided life-support to over 170,000 patients globally (8). ECLS is based on John H. Gibbon's work beginning in the 1930s and culminating with the first successful extracorporeal cardiopulmonary bypass case in 1953 enabling surgeons to perform open-heart operations (7, 40). ECLS utilizes an extracorporeal blood pump coupled with a gas exchange unit to provide cardiopulmonary support for patients with severe cardiac and/or respiratory failure. Venovenous (VV) ECLS provides gas exchange and returns blood to a central vein or the right atrium for respiratory support alone. In contrast, venoarterial (VA) ECLS provides gas exchange and cardiovascular support by returning blood to a central artery for combined respiratory and circulatory support. ECLS is often conventionally referred to as ECMO (extracorporeal membrane oxygenation support). However, newer ECLS technologies do not utilize a “membrane oxygenator” leading to the adoption of the newer more inclusive term of ECLS (41).

Historically, the earliest clinical trial with ECLS failed to demonstrate improved survival in adults (42); however, improved outcomes were observed in neonatal and pediatric patients with respiratory or cardiac failure (43–46). Thus, over the subsequent few decades, ECLS was primarily used to support neonates and pediatric patients with acute respiratory and cardiac failure, including children following cardiac surgery. With improvements in technology and experience, indications for the utilization of ECLS have increased rapidly, and include children and adults with acute respiratory failure and shock, as a bridge to decision when prognosis is uncertain, as a bridge to heart and/or lung transplant, and in rescue of patients with cardiac arrest refractory to cardiopulmonary resuscitation (E-CPR) (3–5, 7, 47, 48). Medical conditions that were once absolute contraindications, such as organ failure following stem cell transplant and chimeric antigen T cell therapy (CAR-T), are now reconsidered for support with ECLS (28, 49). Previously the domain of quaternary care centers, technological advancements have led to an increased number and greater variety of types of centers to offer ECLS. When ECLS is not available at certain locations, mobile ECLS programs have allowed patients to be cannulated locally and transferred to specialized ECLS centers (50).

Outcomes following ECLS vary with patient age and indication for support, ranging from >90% survival in neonates with meconium aspiration syndrome, 65% for all pediatric ECLS indications, 46% in adults with ARDS, to 30% in adults following cardiac arrest (2, 6, 9, 51, 52). While overall survival rates for ECLS patients continue to modestly improve for both adults and children (52), utilization in increasingly complex conditions has led to a plateau in mortality rates for certain conditions, such as cardiac failure in children and neonates (53). When evaluating the potential risks and benefits of ECLS, risk of mortality and ECLS-associated morbidities should be assessed. Serious complications, including bleeding, infection, central nervous system injury, severe deconditioning, and acute kidney injury (54–57) are not uncommon. The impact and potential risk to other patients related to ECLS-associated resource utilization (e.g., intensive care nurses, intensive care beds, blood products) also may need to be considered in the global calculus of risk and benefit of ECLS utilization.

## Clinical Cases

As ECLS technology advances at a rapid pace, there are few technical limitations to offering ECLS as a potentially life saving intervention—therefore, “we *can*.” However, within the context of a wide range of mortality rates, risks for severe morbidities, limited outcome data, and clinician ethical duty to save lives, ECLS clinicians are often faced with the extremely difficult medical and moral decision—“*should* we?” Unfortunately, there is little ethical guidance to assist bedside clinicians in making these complex decisions.

The following hypothetical cases, based on an amalgam of actual clinical cases, highlight several important ethical dilemmas that arise in the provision of ECLS.

### Case 1

*Lily (pseudonym) was a 4-year-old girl found 90 minutes after being submerged in a cold-water lake. Rescuers initiated cardiopulmonary resuscitation at the scene. CPR (cardiopulmonary resuscitation) was provided for 30 minutes en route to the nearest community hospital with return of spontaneous circulation upon arrival. An adult cardiothoracic surgeon had recently established an adult ECLS program at this community hospital. Based on the possibility of neurologic recovery following cold water drowning, the medical team cannulated Lily onto ECLS despite minimal evidence of neurological function. Open chest cannulation was performed given that only adult-size ECLS cannulas were available. The child was then transported to a nearby quaternary academic pediatric center using a portable ECLS team. After rewarming to normothermia, Lily's physical examination revealed lack of neurologic function except for abnormal spontaneous respirations. Head CT revealed diffuse cerebral edema with loss of gray-white matter differentiation, concerning for devastating and likely irreversible neurologic injury. Based on the low likelihood of any neurologic recovery, the family and the medical team jointly decided to compassionately withdraw life support. Lily's parents wished to pursue organ donation and found significant meaning out of this tragedy when her liver and kidneys were successfully transplanted following a circulatory determination of death (DCDD) protocol*.

In this case, urgent medical decision making is complex and challenging when the potential risks and benefits are unpredictable, and there is substantial prognostic uncertainty. ECLS did not provide direct benefit to the child, however the utilization of this intense resource provided benefit to the family and society. The family had additional time to grieve the loss of their child and were given the opportunity to graciously offer the gift of organ donation.

### Case 2

*Betty (pseudonym) is a previously healthy obese unvaccinated 22-year-old woman who was critically ill with severe acute respiratory distress syndrome (ARDS) secondary to SARS-CoV-2 pneumonia complicated by secondary bacterial infection. Despite maximal support with mechanical ventilation, her oxygenation remained quite poor, and she was placed on venovenous ECLS on hospital day five. Due to limited clinical staff and ECLS pumps at the adult hospital during the pandemic, she was transferred from an adult facility to a pediatric ECLS center using a mobile ECLS transport team. While she remained on ECLS, the pediatric intensive care unit (PICU) reached capacity, and critically ill children were diverted to other community-based facilities. After four weeks of ongoing support via ECLS, she was ultimately decannulated. Due to severe deconditioning and lung disease, she remained in the PICU for an additional three weeks during which time a tracheotomy was performed, and she was transitioned to a portable ventilator*.

This case highlights the challenges and consequences of allocation of resource intense therapies during a pandemic. While rapid advancements in technology allow for portable ECLS, transferring this young adult patient to a pediatric facility consequently impacted the ability of the institution to provide specialized pediatric care to other pediatric patients.

## Recommendations For a Research Agenda

Proactive and explicit exploration through collaboration and research of ethical considerations that arise in clinical cases like these may mitigate potential risks and promote: (1) respect for the well-being and autonomy of patients, families, and staff; (2) optimization of patient outcomes while minimizing undue burdens for patients and families; and (3) responsible, equitable, and fair allocation of resources. Navigating complex and challenging questions that arise in ECLS will hopefully lead to improved quality of patient care through a better understanding of the ethical and societal considerations.

To develop a research agenda to inform ethical guidance for clinicians and institutions in the utilization of ECLS, a multi-disciplinary team of pediatric specialists in ECLS, critical care, cardiothoracic surgery, palliative care, and bioethics convened at a single academic institution. Using an iterative consensus process, a broad range of research questions were collected and refined by the group over a series of monthly meetings for approximately 18 months. The broad list was refined by removing duplicates and then selecting questions according to the following criteria: (1) frequency of occurrence in ECLS; (2) uniqueness to ECLS; (3) urgency; (4) feasibility to study; and (5) potential to advance the practice of ECLS and improve quality of patient care. To create the research agenda, refined questions were categorized into the following themes: (1) scope and frequency of ethical dilemmas involving ECLS; (2) clinical decisions and ethical guidance to initiate, forgo, or discontinue ECLS; (3) approaches to optimize communication with and provide emotional support for families and clinical team members; and (4) research design and implementation in ECLS (Tables 1, 2).

**A. Scope and Frequency of Ethical Dilemmas**
  Ethical dilemmas are common in the provision of ECLS, as highlighted by the COVID-19 pandemic. However, few empirical data describe the nature and frequency of ethical issues that arise in ECLS. In order to identify solutions, the problems need to be clearly defined.
  - What ethical dilemmas do clinicians and families experience in ECLS and how frequently do these arise?
**B. Clinical Decisions and Ethical Guidance to Initiate, Forgo, or Discontinue ECLS**
  ECLS decision-making is complex and influenced by a myriad of interacting forces at the bedside, the institution and in society. Decisions may be fraught with challenges and complexity due to the combination of several factors: (1) limited time frame for decision-making; (2) life and death circumstances; (3) large, multidisciplinary clinical teams with potential for differing opinions; (4) prognostic uncertainty; (5) potential for serious complications; (6) increasingly heterogenous patient populations with greater complexity in which ECLS is utilized; and (7) national and international guidelines based on expert opinion rather than clinical data. These challenging circumstances often lead to variability in decisions made by individual or relatively small groups of clinicians potentially resulting in the inconsistent use of ECLS (e.g., variability among diagnoses, patient populations, and geographic regions) (27, 29, 38, 39). As a complex technology requiring a multi-disciplinary team, the quality of ECLS delivered may be variable among centers. To determine if access to high quality ECLS is distributed justly, these forces and the extent of their influence may be explored through the following questions:
**1. Who Decides**
    - What are existing decision-making models for ECLS and how do they lead to variability in use? What does shared medical decision making look like within the context of high urgency and high stakes decisions?
    - What impact does including and excluding various clinical team members from the decision-making process have on: complexity of the decision-making process, quality of medical care, utilization of ECLS, job satisfaction of clinicians, and family support?
    - What role does ECLS-specific training of clinical team members have on the decision-making process?
    - Should ECLS ever be initiated or withdrawn against a family's wishes? If so, what are the morbidity and mortality thresholds for such decisions?
**2. How We Decide**
    - Do clinicians ever limit the use of ECLS based on perceived ethical barriers that are not empirically substantiated? If so, what are the perceived ethical barriers, and how should they be addressed?
    - What biases and assumptions exist among the clinical team, institutions, and organizations that may impact how decisions are made?
    - How are bedside decisions influenced by clinical outcome data, patient comorbidities, and institutional availability of ECLS resources?
    - Why and how does ECLS utilization (initiation, forgoing, withdrawing) vary between institutions or between clinical teams within an institution? What factors contribute to these variations in practices, and how can they be mitigated to ensure equitable care?
    - What factors influence the decision to end life by withdrawing ECLS? How are these decisions made? How are conflicting opinions resolved?
    - How does the urgency of the clinical situation impact clinician decision-making? How does this urgency impact the process of obtaining informed consent from patients and their surrogates?
    - What role, if any, should ECLS center volume and experience play in determining which patient diagnoses are supported by ECLS?
    - How should relatively scarce ECLS equipment and staff be allocated during conventional, contingency, and crisis standards of care?
    - Are there clinical situations when E-CPR should be considered obligatory or prohibited? If so, how are these clinical situations determined and assessed?
    - What is the appropriate level of training and experience needed to determine ECLS candidacy and initiate support? How does the advent of mobile ECLS influence these decisions?
**3. Use of Outcomes Data in ECLS Decision-Making**
    - Are current outcome data adequate to drive or support decision-making in ECLS? What additional outcome data would improve decision-making?
    - How do clinicians' personal values influence interpretation of outcome data and how does this lead to variability in use of ECLS?
    - How do we determine appropriate morbidity and mortality thresholds for compelling or withholding ECLS?
    - Are outcome data commonly used in decision making or are decisions based primarily on clinical experiences? How accurately do clinicians prognosticate?
    - What are the most important long-term outcomes (e.g., physical and psychologic health, cognitive function, social function, quality of life, development and educational) for survivors of ECLS and their families? How should these outcomes influence bedside decision-making in ECLS?
**4. Societal Influence on Bedside Decision-Making**
    - Is ECLS viewed by institutions and society as a conventional therapy (i.e., obligatory) or as an extraordinary intervention (i.e., not obligatory) or both? How does this view influence ECLS utilization at the bedside?
    - How do societal values and beliefs (e.g., values and perceptions of physical and cognitive disabilities, cultural views about life and death, allocation of health care spending) influence the use of ECLS?
    - Does society have an obligation to ensure equitable access to ECLS for all patients? For example, can individual institutions refuse to offer ECLS without obligation to transfer or should referral networks be required? Should ECLS centers of excellence be required to have mobile ECLS teams to improve equitable access?
    - At the societal level, what factors should be included in the calculation of the costs and benefits [e.g., quality or disability-adjusted life years (QALY, DALY), real costs, use of scarce resources such as highly specialized personnel or intensive care beds] of ECLS? How should these factors be prioritized and who determines the prioritization paradigm? How should these analyses affect bedside decision-making?
    - What role should ECLS play in organ donation for patients declared deceased based on neurologic criteria? Should ECLS be utilized to support organ donors who are declared deceased after circulatory death? What are the ethical implications of these practices?
    - How does systemic racism potentially exacerbate health care disparities when considering utilization of ECLS?
**C. Communication and Emotional Support**
  A paucity of literature exists describing communication related to ECLS. The following questions seek to measure the quality of communication and to explore the influence that communication content and style has on patients and families and within the medical team.
**1. Patients and Families**
    Communicating with patients and their families can be difficult in the critical care environment, especially under the stressful circumstances that often surround ECLS (58). Effective communication with ECLS patients and their families is essential for: (1) eliciting patient and family values, preferences, and needs; (2) optimizing patient and surrogate provided permission and informed consent processes; and (3) promoting beneficial psycho-social outcomes. The focus of the following questions is to understand how clinicians communicate with patients and families around ECLS, both assessing the quality of communication and identifying ways to improve communication with patients and their families:
    - How do clinicians communicate with families regarding risks, benefits, alternative options, and outcomes of ECLS prior to initiating ECLS and during support? What are barriers and potential facilitators of optimal communication?
    - How does the urgency of the clinical situation impact communication with families?
    - Is the conventional model for “informed consent” applicable to the initiation of ECLS? Should informed consent be obtained for every ECLS encounter? Are there any modifications to the informed consent process that may improve communication and guide decision making for families?
    - What is the optimal approach to support the family cognitively and emotionally during the decision-making process to initiate ECLS?
    - How can clinicians balance hope and realistic expectations during communication with families when ECLS is initiated and throughout the course of ECLS?
    - How does language used by clinicians impact the experiences of families and their decisions related to ECLS? How do clinicians' views of appropriateness of ECLS use influence their language? For families who speak a language other than English (or other than the primary language of the medical team), how does the use of language interpreters impact the quality and accuracy of communication?
    - What is the role of cultural navigators when navigating complex decisions regarding ECLS? How are cultural barriers that may arise in complex decision making identified and mitigated?
    - What is the balance between resource utilization and family support when a family requests to continue ECLS after the recommendation has been made to discontinue life support?
    - How should clinicians optimally provide psycho-social support for patients and their families both acutely and long-term when ECLS is utilized?
**2. Clinical Team**
    ECLS requires a collaborative and multidisciplinary approach to patient care. Guidelines for ECLS based on expert opinion and experience rather than on clinical data may lead to disparate opinions among clinical team members. Unresolved discord among clinical team members may negatively impact quality of care and patient safety. Understanding and improving communication among clinical team members may help to reconcile these differences. Optimal communication may limit intramural discord, reduce individual moral distress, and thereby improve the quality of patient care. Understanding of the impact of communication among the clinical team requires investigation.
    - How does verbal and non-verbal language used by ECLS teams bias the institutional culture surrounding the “appropriate” use of ECLS? How do institutional cultures and biases affect decision-making in ECLS?
    - How does the interaction and communication among a diverse clinical team impact the experience of patients and their families, team performance, and clinical outcome?
    - How should team leaders manage dissent among clinical team members?
    - How does a lack of consensus among clinical team members affect the quality of patient care, patient emotional well-being, family members, clinical team, unit morale, and other unit patients?
    - Should parents and/or surrogates be made aware of differing opinions within the clinical team? If so, what approach should be used?
**D. Clinical Research Design and Implementation**
  Research in ECLS may be ethically challenging due to the complexity and intensity of the clinical circumstances. Additionally, because ECLS clinicians and researchers are typically one and the same, ambiguity between clinical and research practices of ECLS may exist. These overlapping relationships and the clinical context may unduly influence family expectations, decision-making, and participation in clinical research. Furthermore, designing optimal research studies to advance ECLS may be difficult given the life-threatening context, heterogeneity and complexity of clinical diagnoses, lack of standardized processes, and diversity of clinical opinions about effectiveness. The following questions should be addressed to optimize research practices in ECLS:
  - How do the clinical context and provider relationships influence families' expectations, understanding, and willingness to participate in ECLS research?
  - How do families experience participation in ECLS research?
  - How do researchers ensure that patients and families are optimally protected during ECLS research and improve their experiences?
  - How do provider views about benefit or futility of ECLS in a particular population influence provider endorsement of or participation in research studies in ECLS?
  - In what contexts are randomized trials involving ECLS justified? What alternatives may be appropriate and under what conditions?
  - Should the approach to clinical research in ECLS differ from other “life support therapies”?

**Table 1:** Research agenda outline for ethics in extracorporeal life support.

**A. Scope and Frequency of Ethical Dilemmas**
**B. Clinical Decisions to Initiate, Forgo or Discontinue ECLS**
** 1. Who Decides**
** 2. How We Decide**
** 3. Use of Outcomes Data in ECLS Decision-Making**
** 4. Societal Influence on Bedside Decision-Making**
**C. Communication and Emotional Support**
** 1. Patients and Families**
** 2. Clinical Team**
**D. Clinical Research Design and Implementation**

**Table 2:** Research agenda: example questions to address through normative and empirical research.

**Area of research interest**	**Example research questions**
Scope and Frequency of Ethical Dilemmas	What ethical dilemmas do clinicians and families experience in ECLS?
	How frequently do these ethical dilemmas arise?
Clinical Decisions to Initiate, Forgo or Discontinue ECLS	What are existing decision-making models for initiation, forgoing, or discontinuing ECLS? How are decisions made within the context of high urgency and high stakes decisions?
	How and why does ECLS utilization vary between institutions? What factors contribute to these variations in practices, and how can they be mitigated to ensure equitable care?
	How do clinicians' personal values influence interpretation of outcome data? How are appropriate morbidity and mortality thresholds for compelling or withholding ECLS determined?
	Is ECLS viewed by institutions and society as a conventional therapy or an extraordinary intervention? How do societal values influence ECLS utilization and discontinuation?
Communication and Emotional Support	How does the medical team's approach to communication impact family's understanding, experience and outcomes?
	What are the barriers and potential facilitators of optimizing communication with families?
	How can disagreements and biases among clinicians be minimized through thoughtful discourse? Should surrogate decision makers be made aware of differing opinions within the medical team? If so, what approaches should be used?
Clinical Research Design and Implementation	In what contexts are randomized trials involving ECLS justified? What alternatives may be appropriate and under what conditions?
	Should the approach to clinical research in ECLS differ from other life support therapies, such as mechanical ventilation?

## Conclusions

The *Seattle Ethics in ECLS Consortium* identified and prioritized important ethical questions that warrant further empirical and normative research in the domains of societal decision-making, bedside decision-making, patient/family communication, medical team dynamics, and research ethics. Identifying ethical considerations in the delivery of ECLS is an initial step toward: (1) better understanding the ethical dilemmas encountered, (2) developing approaches to address these ethical dilemmas, and ultimately (3) improving the quality of medical care provided and support for families when utilizing ECLS.

## Data Availability Statement

The original contributions presented in the study are included in the article/supplementary materials, further inquiries can be directed to the corresponding author/s.

## Author Contributions

ML-N founded and led the SEE Consortium and conceptualized the initial manuscript. ML-N and JC co-facilitated the SEE Consortium discussions to develop the ECLS research agenda and drafted, revised, and approved the final manuscript as submitted. JC and JD performed background literature searches. HB, EBo, TB, JD, AD, DM, JR, JT, and BW all participated in the SEE Consortium, vetted the research questions, edited and approved the final manuscript. EBe vetted the research questions, edited and approved the final manuscript. All authors contributed to the article and approved the submitted version.

## Conflict of Interest

The authors declare that the research was conducted in the absence of any commercial or financial relationships that could be construed as a potential conflict of interest.

## Publisher's Note

All claims expressed in this article are solely those of the authors and do not necessarily represent those of their affiliated organizations, or those of the publisher, the editors and the reviewers. Any product that may be evaluated in this article, or claim that may be made by its manufacturer, is not guaranteed or endorsed by the publisher.
